# Cronkhite-Canada Syndrome: Review of the Literature

**DOI:** 10.1155/2013/856873

**Published:** 2013-11-28

**Authors:** Marcela Kopáčová, Ondřej Urban, Jiří Cyrany, Jan Laco, Jan Bureš, Stanislav Rejchrt, Jolana Bártová, Ilja Tachecí

**Affiliations:** ^1^2nd Department of Medicine, Charles University in Praha, Faculty of Medicine at Hradec Králové, University Teaching Hospital, Sokolska 581, 500 05 Hradec Králové, Czech Republic; ^2^Department of Gastroenterology, Vitkovice Hospital, Zalužanského 1192/15, 703 00 Ostrava-Vítkovice, Czech Republic; ^3^The Fingerland Department of Pathology, Charles University in Praha, Faculty of Medicine at Hradec Králové, University Teaching Hospital, Sokolska 581, 500 05 Hradec Králové, Czech Republic

## Abstract

Cronkhite-Canada syndrome is a rare disease characterised by diffuse polyposis of the gastrointestinal tract, diarrhoea, weight loss, abdominal pain, cutaneous hyperpigmentation, dystrophic changes of fingernails, and alopecia. The etiology is probably autoimmune and diagnosis is based on history, physical examination, endoscopic findings of gastrointestinal polyposis, and histology. The disease is very rare; about 450 cases have been described in the literature so far. We present a review of the literature with our own picture documentation of this rare condition.

## 1. History 

Cronkhite-Canada syndrome (CCS) is a rare disease; about 450 cases have been described in the literature so far. The disease was first described in 1955 by the American internist Leonard Wolsey Cronkhite and the American radiologist Wilma Jeanne Canada in the New England Journal of Medicine. They published two cases of an unusual fatal syndrome of diarrhoea, nausea, vomiting, and abdominal pain in a 42-year-old female and a 75-year-old female. Several weeks prior to the symptoms, loss of hair, eyebrows, and axillar hair with diffuse brown discoloration of the face, neck, and hands, atrophic tongue with brown discoloration, and onychodystrophy were observed. Anaemia in laboratory examination and gastrointestinal polyposis were found. Gastric and colonic histology was consistent with benign adenomatous polyposis. The oesophagus was normal [1]. 

Jarnum and Jensen [2] established the term Cronkhite-Canada syndrome in their publication in 1966. They published a case report with two new observations in CCS patients: protein-losing enteropathy with electrolyte disturbances (hypocalcaemia, hypomagnesaemia, and hypokalaemia) and presence of nonadenomatous cystic polyps [2].

In 1972, Johnson et al. [3] published that the polyps in the stomach and large intestine are hamartomas and confirmed the description of Jarnum and Jensen.

Goto divided the disease into five groups according to the leading symptom in 1995 [4]; type 1: diarrhoea is dominant, type 2: dysgeusia, type 3: abnormal sensation in the mouth with thirst, type 4: abdominal symptoms other than diarrhoea, and type 5: alopecia as a main symptom. All patients must have gastrointestinal polyposis and hyperpigmentation.

The estimated incidence of CCS is one per million according to the study performed by Goto, the largest study on CCS with 110 patients [4, 5]. The mean age of onset is estimated to be in the fifth to sixth decade with a slight male predominance in the ratio 3 : 2 [6]. 

## 2. Etiology and Clinical Features

The etiology of CCS is currently unknown. So far, there is no strong evidence to suggest a familial predisposition. The disease is sporadic, so hereditary origin is not supposed. Etiology is probably autoimmune, but infectious cause was also considered because of inflammatory cell infiltration with mononuclear cells and eosinophils [7]. Cases have been associated with elevated antinuclear antibody (ANA) and IgG4 levels [8, 9]. IgG4-related autoimmune disease is a recently described multisystem disorder characterised by IgG4 plasma cell infiltration with manifestations including autoimmune pancreatitis, sclerosing cholangitis and retroperitoneal fibrosis. Some sporadic juvenile CCS polyps were studied by Riegert-Johnson et al. with findings of infiltration with IgG4 plasma cells.

Regardless of whether the IgG4 plasma cell infiltration of CCS polyps (reported by Riegert-Johnson et al.) is linked to IgG4-related autoimmune disease or not, this finding is the first clue to the pathophysiology of CCS [10]. Immunostaining for the autoimmune-related IgG4 antibody is significantly increased in CCS polyps compared to other diseases and normal control tissues. Furthermore, immunosuppression by corticosteroids or long-term azathioprine may eradicate or lessen manifestations of CCS. These histological findings and treatment responses are consistent with an autoimmune mechanism underlying CCS [11].

There is also an association between CCS and hypothyroidism and various other autoimmune diseases such as membranous glomerulonephritis, systemic lupus erythematosus, rheumatoid arthritis, and scleroderma. Mental and physical stress has been confirmed to be among the most important risk factors for this syndrome [6, 8, 12]. Familial incidence has been described only once, in two members of one family [13].

**Figure 1:**
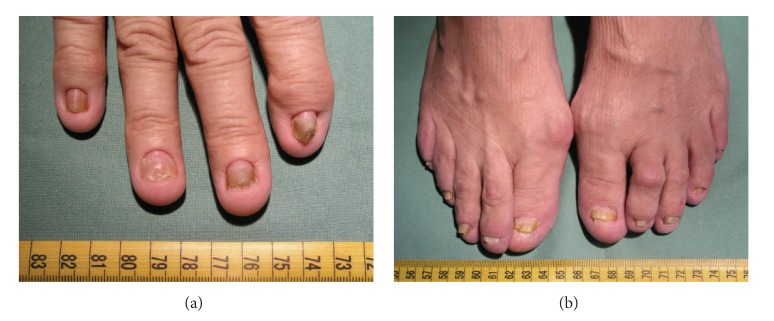
Atrophic changes of fingernails, hands, and feet.

**Figure 2:**
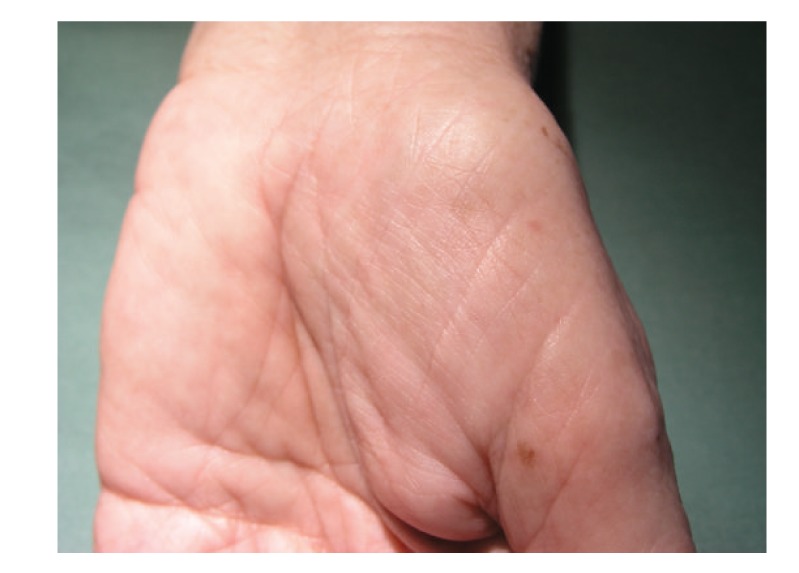
Pigment dots on palms.

**Figure 3:**
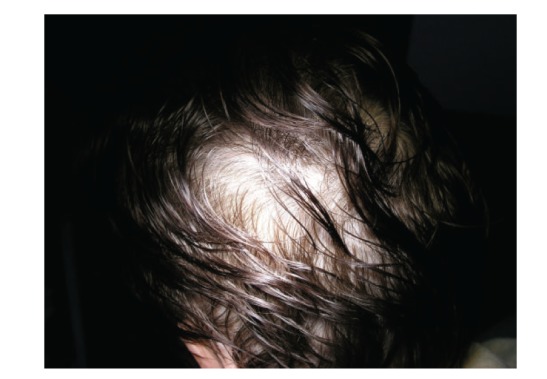
Diffuse alopecia.

Diagnosis is based on history, physical examination, endoscopy with finding of gastrointestinal polyposis, and histology. CCS is characterised by diffuse multiple polyps of the gastrointestinal tract, diarrhoea, weight loss, abdominal pain, cutaneous hyperpigmentation, dystrophic involvement of fingernails, and alopecia (Figures 1(a), 1(b), 2, and 3). Other symptoms such as hypogeusia and xerostomia have also been described in the literature [6]. Dysgeusia can be caused by mucositis, oral infections, and other abnormalities of mucosal surface. Zinc and copper deficiencies are also believed to cause hypogeusia in some patients [14].

Protein-losing enteropathy is often observed. Polyps are frequent in the stomach and small and large intestine but do not occur in the oesophagus. The gastric mucosa can be thickened (hypertrophic gastric folds mimic Menetrier's disease in some cases), but it can be atrophic with polypoid lesions in others [6, 12, 15].

**Figure 4:**
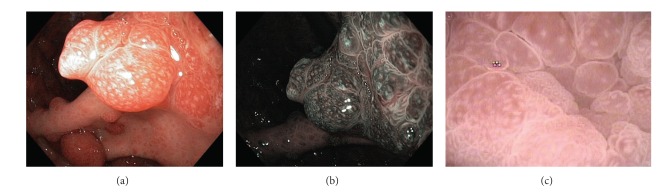
Stomach. Strawberry-like polyps, (a) high resolution white light endoscopy, (b) narrow band imaging (NBI), (c) zoom.

Gastroscopy (Figure 4) shows red and edematous granular polyps (strawberry-like) with giant mucosal folds (carpet-like polyposis of the stomach). On confocal laser endomicroscopy, hyperplastic mucosa and hyperplastic polyps are detected (Figure 6). Similar polyps could be found in the duodenum (Figures 7 and 8). Some small denuded areas without villi are seen in the small intestine (Figure 9). Duodenal mucosa is swollen with nodulations and sparse villi. Villi in the jejunum are clearly visible, but there are areas without villi in the jejunum usually on the top of the folds. Colonic polyps have been characterised as sessile and could be “strawberry-like” according to some studies (Figure 12) [16–19].

Seventy-five percent of all CCS cases reported in the global literature have been reported from Japan. Coincident gastric cancer occurred in 10% of the cases. The rate of coincident gastric cancer among CCS patients is significantly higher than the prevalence of gastric cancer in the general Japanese population [4].

According to Chinese retrospective meta-analysis of 20 years (1985–2006), there were only 35 cases of CCS in the whole of China [18]. There has been no special occupation associated with an increased incidence of CCS. Hypogeusia is the dominant initial symptom which is usually followed by diarrhoea and ectodermal changes including alopecia, nail dystrophy, and skin pigmentation. Gastrointestinal polyposis is closely related to the malabsorption which induced these ectodermal changes. However, there are a small number of cases in which alopecia precedes diarrhoea in the course of the disease [4]. An electrogastrography detects severe gastric myoelectric disorder (Figure 16).

## 3. Histopathology

The histological specimens from stomach and small and large intestines show typical features of benign juvenile-like or hamartomatous polyps, mild infiltration of inflammatory cells including eosinophils, massive submucosal edema mostly located in the lamina propria, hyperplasia of the foveolar epithelium, focal hyperplastic features, and cystic dilation of the mucosal glands. In approximately half of the patients, some polyps reveal adenomatous changes with stromal edema and dilated glands. “Conventional” adenomas, juvenile-like polyps, and serrated adenomas, whose crypts show a saw-toothed growth pattern with possible dysplastic changes, were described [20, 21].

Microscopic examinations reveal a significant mucosal alteration in all nonpolypoid biopsies, which are more pronounced in small bowel samples. In general, it comprises impaired architecture of crypts, including dilation and branching, edema, and presence of mixed inflammatory infiltrate (Figure 10). The latter is composed mainly of lymphocytes, plasma cells, and eosinophils, with scattered neutrophils. Interestingly, the surface of duodenal, jejunal, and ileal mucosa is rather flat due to subtotal and/or total atrophy of villi (Figure 10).

**Figure 5:**
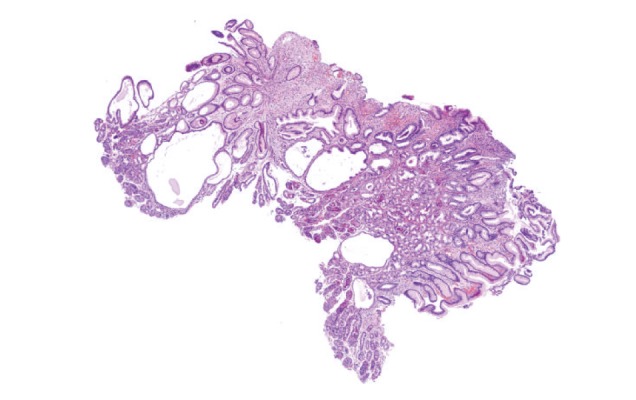
Juvenile polyp of gastric mucosa. Foveolar hyperplasia and dilation of glands are evident (hematoxylin-eosin, original magnification 4x).

**Figure 6:**
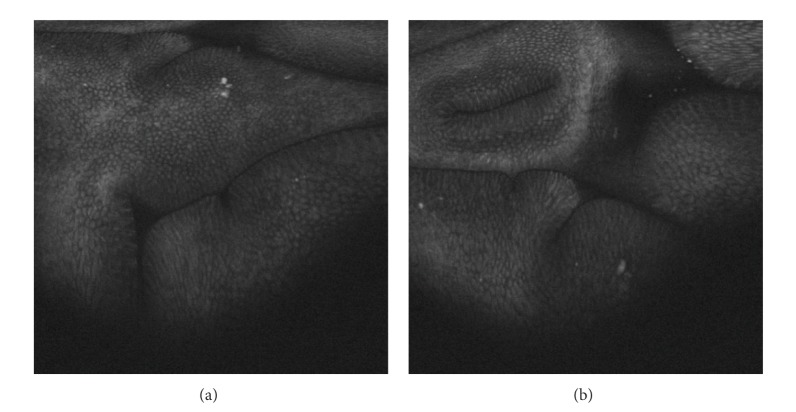
Stomach. Hyperplastic crypts in confocal laser endomicroscopy (original magnification 1000x).

**Figure 7:**
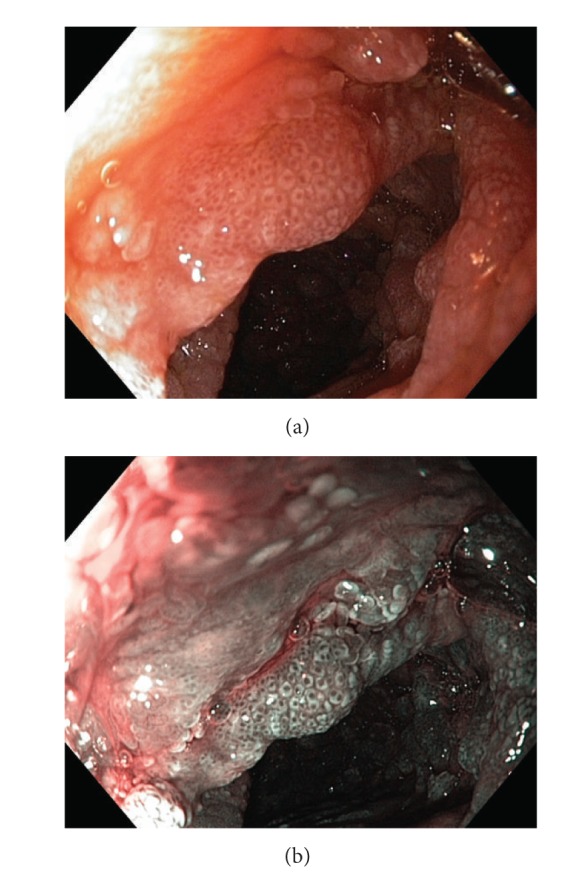
Duodenum. Atrophic changes with multiple strawberry-like polyps, high resolution white light endoscopy, and narrow band imaging (NBI).

**Figure 8:**
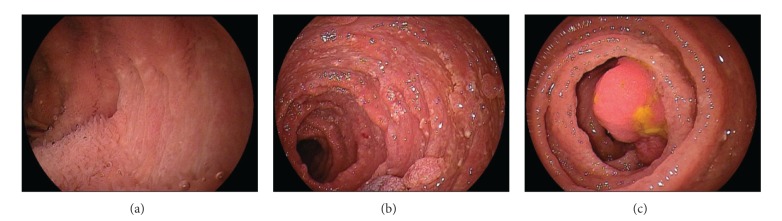
Jejunum. (a) Atrophic part of the jejunal mucosa without villi-denuded areas, (b) small hyperplastic/juvenile polyps of the jejunum, (c) large stalked polyp.

**Figure 9:**
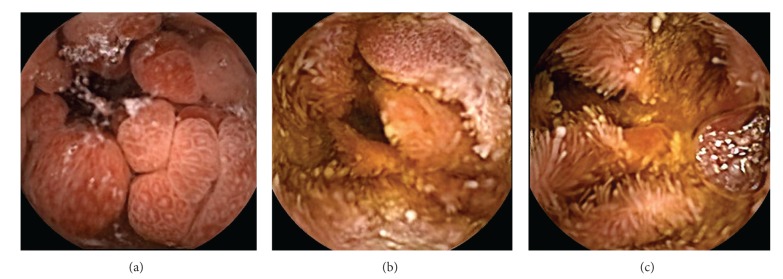
Capsule enteroscopy. (a) Strawberry-like polyps in the jejunum, (b) (c) multiple denuded areas without villi in the jejunum.

**Figure 10:**
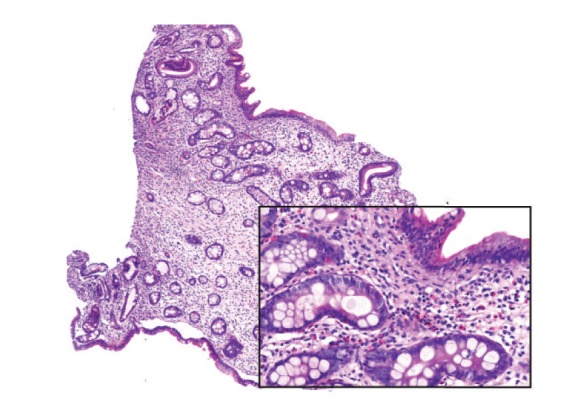
Nonpolypoid jejunal mucosa shows irregularly shaped crypts, edema, and inflammation (hematoxylin-eosin, original magnification 6x). Inset: flattened mucosal surface without villi (hematoxylin-eosin, original magnification 20x).

**Figure 11:**
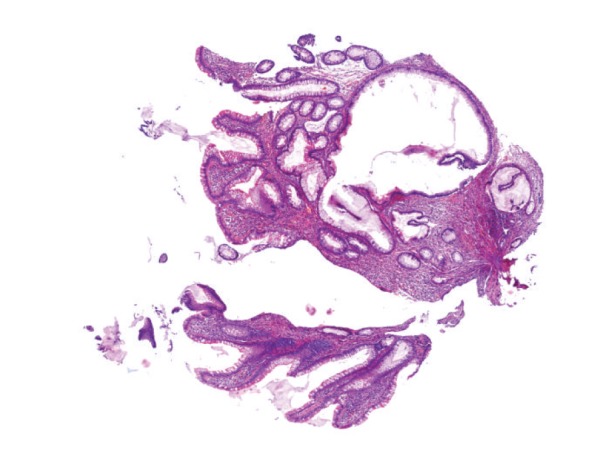
Juvenile polyp of jejunal mucosa shows shortening of villi, dilation of crypts and mild inflammation (hematoxylin-eosin, original magnification 5x).

**Figure 12:**
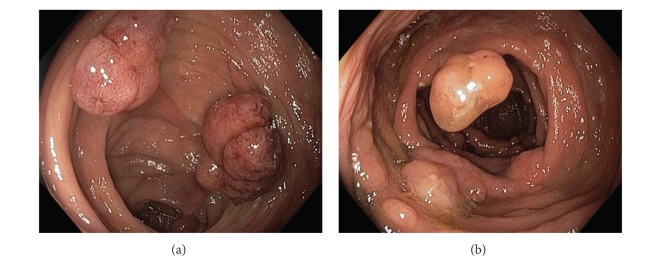
Multiple colonic polyps; juvenile polyps/hamartomas, tubular adenomas, traditional serrated adenomas.

**Figure 13:**
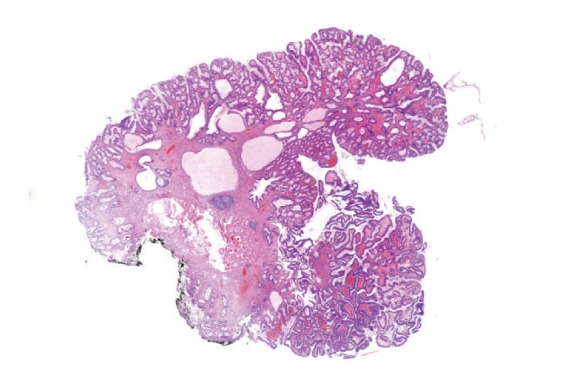
“Conventional” tubular adenoma of the colon with low-grade dysplasia, with focal dilation of crypts (hematoxylin-eosin, original magnification 2x).

**Figure 14:**
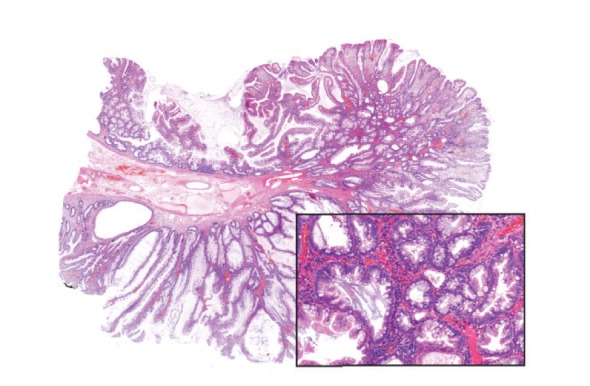
Traditional serrated adenoma of the colon with low-grade dysplasia (hematoxylin-eosin, original magnification 2x). Inset: serrated morphology and eosinophilic cytoplasm of tumour cells are evident (hematoxylin-eosin, original magnification 10x).

**Figure 15:**
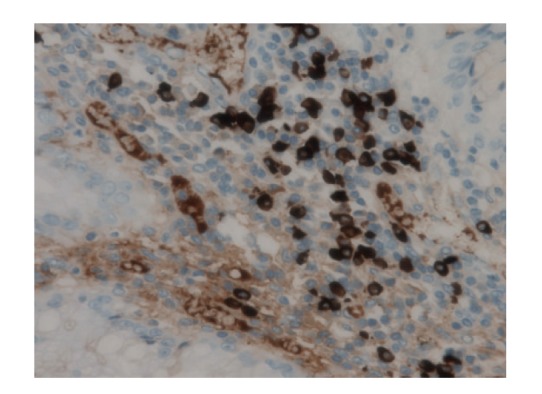
Numerous IgG4-positive plasma cells in colonic traditional serrated adenoma (immunohistochemistry IgG4, original magnification 400x).

**Figure 16:**
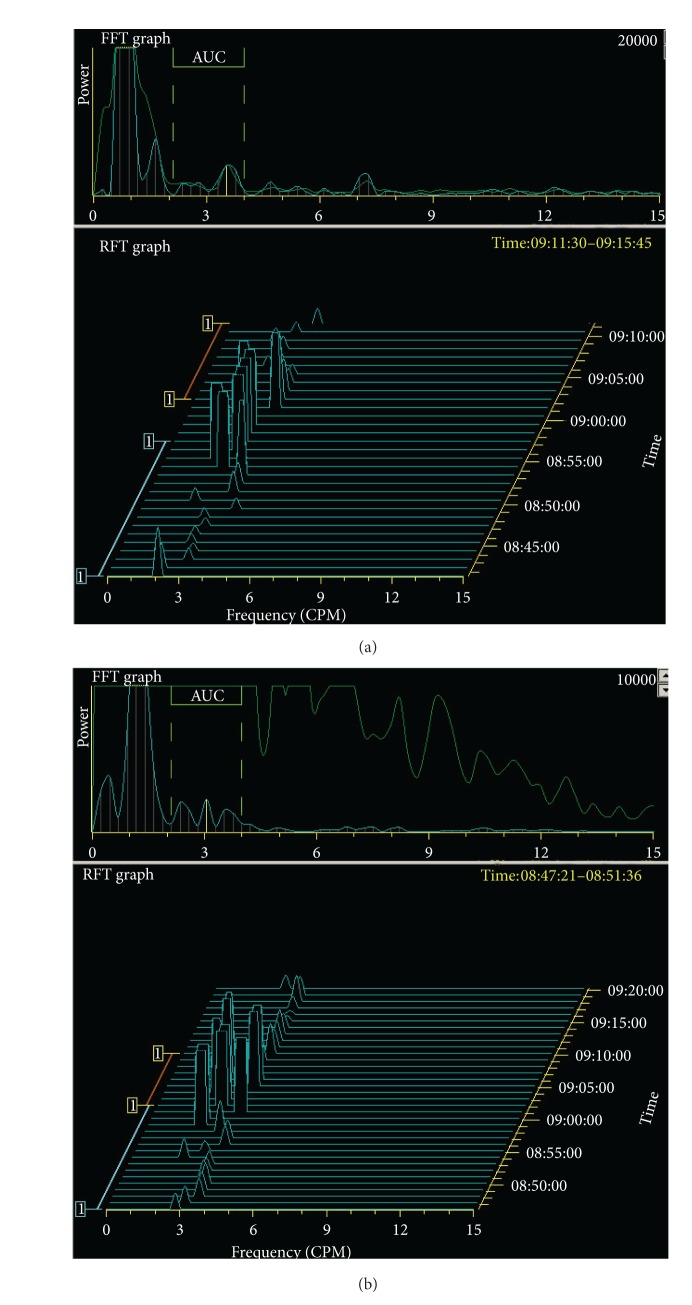
Electrogastrography. Severe gastric arrhytmia (running spectral analysis based on Fourier transform).

Microscopically, gastric (Figure 5), duodenal, jejunal, and ileal (Figure 11) polyps and some colonic polyps have an appearance similar to that of juvenile/hamartomatous polyps without dysplastic changes. The remaining colonic polyps are diagnosed either as “conventional” tubular adenomas (Figure 13) or traditional serrated adenomas without high-grade dysplasia (Figure 14). In immunohistochemical investigations, the presence of CD138-, IgG-, and IgG4-positive plasma cells is noticed (Figure 15).

## 4. Complications and Prognosis

Potentially fatal complications, such as malnutrition, gastrointestinal bleeding, and infection, often occur with a mortality rate of more than 50% [7].

Common complications are gastrointestinal bleeding with anaemia, intussusception, and rectal prolapse [12]. Some uncommon complications and concomitant diseases have been reported in the literature: recurrent severe acute pancreatitis [22], myelodysplastic syndrome [23], giant cell bone tumour [24], multiple rib fractures [17], cecal intussusception in an adult patient [25], schizophrenia [26], portal thrombosis, and membranous glomerulonephritis [8].

Gastric and colonic cancers are common complications. Nests of cancer cells were described in the gastric mucosa by Egawa et al. [27] which were predominantly poorly differentiated with tubular formation. No adenomatous changes were noted around the cancer cells. This suggested that the cancer originated from the gastric mucosa without adenoma-carcinoma sequence. To validate this theory, Egawa et al. stained the tissue for Ki-67 and p53. Only cancer cells had overexpression of these proteins, so only these cells had the ability to proliferate without cellular control [27]. Adenomas were reported in CCS patients in the colon with potential transition to carcinoma. Microsatellite instability and overexpression of the p53 protein were found in the cancer lesions and serrated adenoma lesions. None of the lesions showed a loss of heterozygosity of various genes or K-RAS mutations [20, 28]. Zügel et al. [29] reported a 63-year-old lady with Cronkhite-Canada syndrome who developed colorectal cancer. A hemicolectomy was performed, and the tumour specimen was prepared for DNA analysis and immunohistochemical screening. They found a mutation of p53 gene without APC- and ras-gene alteration and expression of ErbB2 proto-oncogene. The steps of mutation do not follow the adenoma-carcinoma sequence first described by Vogelstein in 1988 [30]. This and previous observations suggest that carcinogenesis in Cronkhite-Canada syndrome follows another independent sequence [29].

About 15% of CCS patients develop malignancies as CCS may be a premalignant condition for gastric cancer, as well as for colorectal cancer. Periodic examination of the stomach, colon, and rectum is suggested for patients with this syndrome [27]. Due to the rarity of the disease, optimal screening protocols have not been developed, although annual endoscopic surveillance has been widely practiced. Multiple biopsies should be taken in order to identify dysplastic and adenomatous epithelium. Total gastrectomy is indicated in the event of dysplastic changes. According to colorectal carcinoma,the procedure of choice is determined by the location of the lesion. If the colon is carpeted with polyps, subtotal or total proctocolectomy would be indicated [6, 9, 11, 12, 31, 32].

Prognosis of the patient with CCS is poor with a 5-year mortality rate 55%. Spontaneous regressions, however, were observed in 5–10% of CCS cases, regardless of treatment [26].

It was originally thought that the epidermal changes were secondary to profound malnutrition as a result of protein-losing enteropathy. Recent findings have called this hypothesis into question; specifically, the hair and nail changes may not improve with improved nutrition [12].

## 5. Treatment

The optimum treatment of CCS is currently unknown due in part to its rarity. Nutritional support, electrolytes, mineral and vitamin supplementation are necessary but can rarely lead to complete remission. The current literature favours combined therapy based on parenteral nutrition, antibiotics, and corticosteroids [6]. Total parenteral nutrition is preferred to enteral nutrition because of the supposed effect of bowel rest. Other therapies such as antihistamine receptor agonist agents and cromolyn sodium have also been used as a supplementary therapy in patients where degranulating eosinophils and mast cells are found in biopsies [6, 33]. Because of the apparent autoimmune features of the disease, azathioprine and tacrolimus were given to the patients in some cases [34]. Most studies recommend treatment with proton pump inhibitors (or H2 receptor antagonists in older papers). In one case report [35], acute gastritis was found in histology in a *Helicobacter pylori*-positive patient. The patient was given eradication therapy (clarithromycin, amoxicillin, and lansoprazole) resulting in negative C13-urea breath test. Complete remission including remission of polyposis was achieved eight months later [35]. Eradication of *Helicobacter pylori* is recommended also in other papers [24]. In other patients, *Helicobacter pylori* was negative at the time of diagnosis [21]. An anti-TNF-*α* therapy was considered in one paper because of strong intracellular expression of TNF-*α* in the small intestinal mucosa. Unfortunately, an experimental anti-TNF-*α* treatment could not be introduced because of rapid progression of the disease; the patient died within 4 months after the diagnosis was established [16].

Mesalazine therapy is recommended according to one paper [36] and antiplasmin tranexamic acid according to another [26].

Optimum therapy for CCS is not known but several treatment options have been described. Nutritional support, antibiotics, systemic glucocorticosteroids, anabolic steroids, histamine-receptor antagonists, and surgical treatment have all been used with varying degrees of success. Unfortunately, controlled therapeutic trials have not been possible because of the rarity of the disease. Most recently, a combination regimen using histamine-receptor antagonists, cromolyn sodium, prednisone, and suppressive antibiotics has been described. The reported treatment options and rates of success were reviewed [37].

The total treatment period is also unknown; recommendations range from 6 to 12 months of combined therapy. 

We use oral treatment with omeprazol (20 mg twice a day), prednisone (20 mg a day) and azathioprine (2.5 mg per kg/day if a gene for thiopurine s-methyltransferase-TPMT is without mutations). Pancreatic enzymes are given to the patient with bigger meals 3-times a day.

## 6. Differential Diagnosis

CCS often has characteristic features. Usually, it is not difficult to distinguish CCS from other polyposis syndromes, as each exhibits its own characteristic clinicopathology.

Other conditions consisting of multiple hamartomatous polyps of the digestive tract include Peutz-Jeghers syndrome, juvenile polyposis, familial adenomatous polyposis, hyperplastic polyposis, and Cowden disease [9, 38].

Peutz-Jeghers syndrome is an inherited polyposis disorder characterised by hamartomatous polyps and pigmented macules on the lips, buccal mucosa, and skin that usually occur prior to 30 years of age.

Juvenile polyposis develops before 10 years of age and is characterised by hamartomatous polyps with an inflammatory component mostly in the colon. From a histological point of view, this is the main differential diagnosis of the disease. Both CCS and juvenile polyposis are distinguished by juvenile polyps. The difference is in the surrounding mucosa, normal histological appearance in juvenile polyposis, and severe changes of architecture in CCS.

Adenomatous polyposis is an inherited syndrome with an abnormal autosomal dominant gene leading to multiple adenomatous polyps in the colon, progressing to colonic cancer in 100% of the cases by the time of 50 years of age [21].

In hyperplastic polyposis syndrome, the polyps are found in abundance throughout the colon in the absence of gastric or small bowel involvement. Diagnostic criteria are clearly articulated: five or more hyperplastic polyps proximal to the sigmoid colon, two of which are over 1 cm; any number of hyperplastic polyps proximal to the sigmoid colon in patients who have first-degree relatives with hyperplastic polyposis; or more than 30 hyperplastic polyps throughout the colon [39].

Cowden disease is an autosomal dominant disorder with hamartomatous polyposis and extraintestinal manifestations (facial trichilemmomas, macrocephaly, mucocutaneous lesions, acral keratoses, and thyroidal and breast diseases) [21].

## 7. Conclusion 

CCS is a rare and serious disease with a high mortality rate. Improvement of the approach with complex medical therapy and increased knowledge of the disease have led to better prognosis of patients in comparison with former case reports. Etiology seems to be moving towards an autoimmune nature but further research of CCS etiology and treatment is badly needed.
